# Measurement of Ankle Joint Movements Using IMUs during Running

**DOI:** 10.3390/s21124240

**Published:** 2021-06-21

**Authors:** Byong Hun Kim, Sung Hyun Hong, In Wook Oh, Yang Woo Lee, In Ho Kee, Sae Yong Lee

**Affiliations:** 1 Department of Physical Education, Yonsei University, Seoul 03722, Korea; bh_kim@yonsei.ac.kr; 2 International Olympic Committee Research Centre Korea, Yonsei University, Seoul 03722, Korea; 3 Department of Sports Industry Studies, Yonsei University, Seoul 03722, Korea; hyun-ny-love@hanmail.net; 4 Department of Mechanical Engineering, Yonsei University, Seoul 03722, Korea; inwookoh@yonsei.ac.kr (I.W.O.); ywlee0305@naver.com (Y.W.L.); kee.inho0@gmail.com (I.H.K.); 5 Institute of Convergence Science, Yonsei University, Seoul 03722, Korea

**Keywords:** validation, kinematic, inertial measurement units, motion analysis, gait

## Abstract

Gait analysis has historically been implemented in laboratory settings only with expensive instruments; yet, recently, efforts to develop and integrate wearable sensors into clinical applications have been made. A limited number of previous studies have been conducted to validate inertial measurement units (IMUs) for measuring ankle joint kinematics, especially with small movement ranges. Therefore, the purpose of this study was to validate the ability of available IMUs to accurately measure the ankle joint angles by comparing the ankle joint angles measured using a wearable device with those obtained using a motion capture system during running. Ten healthy subjects participated in the study. The intraclass correlation coefficient (ICC) and standard error of measurement were calculated for reliability, whereas the Pearson coefficient correlation was performed for validity. The results showed that the day-to-day reliability was excellent (0.974 and 0.900 for sagittal and frontal plane, respectively), and the validity was good in both sagittal (*r* = 0.821, *p* < 0.001) and frontal (*r* = 0.835, *p* < 0.001) planes for ankle joints. In conclusion, we suggest that the developed device could be used as an alternative tool for the 3D motion capture system for assessing ankle joint kinematics.

## 1. Introduction

The ankle joint is the most frequently involved in human lower body movements, and it plays a vital role in supporting body weight by distributing gravitational and inertial loads. Once injuries, such as strain or sprain, by an external force occur in the ankle joint, they cause deformities in its structure. Impairments of the ankle joint can result in chronic ankle instability; therefore, irregular loading on one side could provoke pain on the ankle. Ankle sprains are common injuries in the general population, as well as among professional athletes [1,2,3]. The characteristics range from structural deficits such as joint laxity to functional impairments in gait [4]. In terms of rehabilitation, measuring the ankle joint movement pattern during ambulating or running can help clinicians determine the optimal care level a patient should receive.

Many clinical settings for gait training and rehabilitation in patients with motor impairments use a three-dimensional (3D) motion capture system considered the gold standard measurement of joint kinematics [5,6]. The 3D motion capture system is one of the measurement tools with high accuracy, e.g., mean absolute marker-tracking errors of 0.15 mm during static trials [6] and 0.2 mm (with corresponding angle errors of 0.3) during dynamic trials [7]. A VICON system, showing high validity and reliability in measuring joint kinematics, has been used as a suitable comparison tool to examine whether alternative systems, e.g., inertial measurement unit (IMU)-based systems, provide a sufficiently accurate method for motion analysis [8,9]. Although this sophisticated system allows the assessment of kinetic and kinematic data from complicated human movements, it has several limitations. The fact that the 3D motion capture system is a marker-based system requiring many cameras is considered the primary limitation. The high cost of the instrument makes it impractical to use in various settings, such as a clinic, the field, or patients’ homes. Furthermore, the system cannot be used to measure and track movements simultaneously.

To overcome the limitations of the 3D motion capture system, many efforts to develop a device that can be simply conducted with a concise process have been made by researchers. Recently, IMUs—a markerless motion capture technology—have been developed as an alternative measurement tool to 3D motion capture devices. An IMU is a wearable-designed device that allows motion measurement data to be sent to a computer in real time and immediate feedback (5) to be received. It collects 3D data (x, y, and z) using a combination of accelerometers, gyroscopes, and magnetometers; it is lighter, smaller, and easier to use than the 3D motion capture system. Collecting and combing raw data from multiple individual sensors are enabled by sensor fusion algorithms, and thus, the estimation of 3D spherical coordinates and Euler angles in a global reference domain can be made [10].

IMUs have been evaluated and shown to be promising in estimating the angular kinematics of lower limb joints, including the hip, knee, and ankle [11,12,13], as well as upper body posture [14]. However, as IMUs are not easily available to all professionals, due to movement complexity, sensor placement, biomechanical model, and calibration procedure that could increase the risk of error of the measurement, most researchers have tried analyzing the movements of joints conducted in the sagittal plane, such as flexion, extension, and hyperextension movements.

Especially, the errors of measurement values for the ankle in the transverse and frontal planes for gait analysis were large, which might be due to the small range of motion in these planes or the differences in the anatomical or biomechanical definitions between the two systems [15]. However, to the best of our knowledge, the number of previous studies that have investigated the angular kinematics of the ankle joint are limited, and it is necessary to establish validity as a clinical tool to aid in the diagnosis of gait impairment and treatment. Therefore, the purpose of this study was to verify whether the newly developed device can be simply operated with a high accuracy and concise calibration process.

## 2. Materials and Methods

### 2.1. Participants and Data Collection

Ten healthy male participants of 30.2 ± 5.3 years, 171 ± 15.3 cm, and 73.6 ± 12.4 kg body mass were recruited in this study. Exclusion criteria for the study were as follows: individuals who had ankle surgery or nervous system damage or disorder and those with any injuries to the lower limbs within the past three months that could affect the neuromuscular function. The study protocol was approved by the Office of Research Ethics at Yonsei University (IRB No. 7001988-202101-HR-1076-03) and all subjects provided an informed consent, which were compliant with the Declaration of Helsinki.

### 2.2. IMU System and Sensor Placements

A Raspberry Pi 3 Model B+ computer and two Adafruit BNO055 IMU sensors were used (Adafruit, New York, NY, USA) for data collection. Sensor data were collected at a constant frequency of 100 Hz. One IMU sensor was placed on top of the instep of the right foot (Sensor 1), and the second IMU sensor was tightly fixed on the right shin (Sensor 2) using a specially designed holder. The part of holder meeting the right shin has a round shape so it does not move horizontally. The sensors were required to be perfectly parallel to each other, as well as the ground, for accurate calculations. Thus, we designed a shoe mount for Sensor 1 and a sensor holder with a strap for Sensor 2. Both parts comprised a set of an acrylic sensor slide plate and acrylic holder for easy detachment during the calibration process. In addition, we mounted four-corner leveling systems on both holders for leveling. Sensor 2 was fixed as reference coordinates by built-in coordinates (ref-coordinates) of the BNO055 IMU sensor. Each sensor was directly wired to the single board computer using 3,4 buses for I2C communication. This whole setup was powered by a portable lithium battery with a capacity of 5000 mAh, lasting more than 3 h, which is sufficient for most IMU-based trainings. Eulerian displacements were calculated by subtracting Sensor 1 coordinate data (test-coordinate) from ref-coordinate values. Displacement values of each axis were referred to the yaw, pitch, and roll status.

### 2.3. Vicon System and Marker Placement

**Figure 1:**
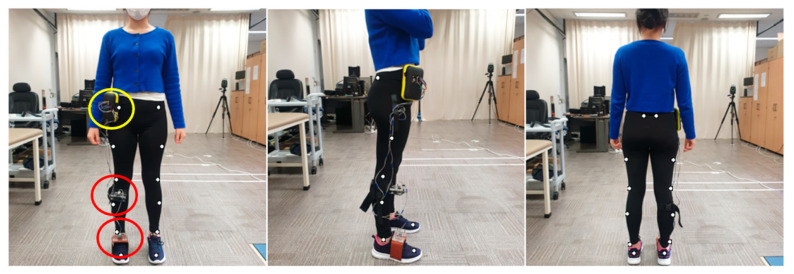
Participants’ setup.

The kinematic data were collected at 100 Hz and their positions targeted the capture volume. The calibration of the Vicon system was conducted before each data collection. The Plug-in-Gait (PiG) lower body model was used to analyze movement at the ankle joints. A total of 16 reflective markers were placed on the participants before testing, and a static calibration trial was initially collected to form a musculoskeletal model based on (Figure 1) an 8-camera motion analysis system (VICON, Oxford, UK). The place of markers was attached to the following landmarks: ASIS, PSIS, mid-lateral thigh, lateral knee joint line, lateral mid-shank, lateral malleoli, calcaneal tuberosity, and head of the second metatarsal.

The participants’ specific information of weight, height, ankle width, knee width, and leg length were measured in the lower body model. Figure 1 shows the participants’ setup of the anterior, lateral, and posterior views with the markers in place. The PiG model of Vicon was used to evaluate all parameters. The lower body was modeled as seven segments (one pelvis, two thighs, two shanks, and two feet). A normal gait cycle was defined from the initial heel-to-heel contact with the same limb.

### 2.4. IMU Joint Angle Calculations

The proposed IMU sensor includes internal algorithms to calibrate the gyroscope, accelerometer, and magnetometer inside the device. The calibrations of gyroscope, accelerometer, and magnetometer were conducted at the same time the investigator held the device with their hand and shook it in the shape of 8. However, the IMU sensor did not contain any internal electrically erasable programmable read-only memory, so we had to perform the formal calibration process every time the device started up.

After the calibration, raw sensor orientation data were received as types of quaternions. These quaternions needed to be converted to a Euler angle, commonly used units, for easy comprehension. Euler angles were obtained from the quaternions via the following equations [16]:

$$\left[\begin{array}{c} \varphi \\ \theta \\ \psi \end{array}\right]=\left[\begin{array}{c} arctan\frac{2\left(q_{0}q_{1}+q_{2}q_{3}\right)}{1-2\left(q_{1}{}^{2}+q_{2}{}^{2}\right)}\\ \arcsin\left(2\left(q_{0}q_{2}-q_{3}q_{1}\right)\right)\\ arctan\frac{2\left(q_{0}q_{3}+q_{1}q_{2}\right)}{1-2\left(q_{2}{}^{2}+q_{3}{}^{2}\right)} \end{array}\right],\;\begin{array}{c} q_{0}=q_{w}=cos\left(\alpha /2\right)\\ q_{1}=q_{x}=sin\left(\alpha /2\right)\text{ cos}\left(\beta_{x}\right)\\ q_{2}=q_{y}=sin\left(\alpha /2\right)\text{ cos}\left(\beta_{y}\right)\\ q_{3}=q_{z}=sin\left(\alpha /2\right)\text{ cos}\left(\beta_{z}\right) \end{array}$$

where φ,θ, and ψ are Euler angles and $q_{0},q_{1},\;q_{2},\;\mathrm{and}\;q_{3}$ are quaternions. α is a simple rotation angle and $\cos\left(\beta_{x}\right)$, $\cos\left(\beta_{y}\right)$, and$\;\cos\left(\beta_{z}\right)$ are the direction cosines (Euler’s rotation theorem).

$$\begin{array}{l} Sensor\;1\;Euler\;angle\\ S_{1}=[\varphi_{1},\;\theta_{1},\;\psi_{1}]\\ Sensor\;2\;Euler\;angle\\ S_{2}=[\varphi_{2},\theta_{2},\;\psi_{2}]\\ Static\;Euler\;angle\\ S_{st}=[\varphi_{st},\theta_{st},\;\psi_{st}]\\ Ankle\;joint\;angle\\ =[\varphi_{1}-\varphi_{2}+\varphi_{st},\;\;\;\theta_{1}-\theta_{2}+\theta_{st},\;\;\;\psi_{1}-\psi_{3}+\psi_{st}] \end{array}$$

**Figure 2:**
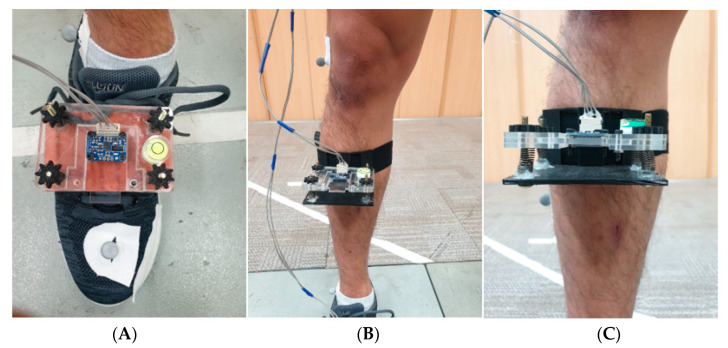
Sensor placement: (**A**) Sensor 1, (**B**) Sensor 2, and (**C**) Sensor 2 holder with strap and leveling.

The coordinate system of the IMU sensor was aligned parallel to the floor, and the angle started at 0° based on that state. As the sensors and ground were started parallelly (Figure 2), ankle motion was generated by simply subtracting Sensor 1 (ref-coordinate) and Sensor 2 (test-coordinate) angles. The static angle value was added to the subtracted value of the IMU sensor.

Description of the location of each IMU sensor (red), Raspberry Pi (yellow), and PiG body model marker location for the: (left) anterior view; (middle) lateral view; and (right) posterior view.

### 2.5. Vicon Joint Angle Calculations

Kinematics of the ankle joint were measured using the Vicon PiG model. Sagittal plane motion of the ankle was taken between the shank anterior to posterior axis and the projection of the axis formed by the heel and toe markers into the sagittal plane of the foot.

Furthermore, frontal plane motion of the ankle was taken between the ankle medial to lateral axis, and the projection of the axis formed both malleoli.

Additional information of the PiG angle calculations can be found on Vicon’s website.

### 2.6. Experiment Protocol

To evaluate the validity between VICON and IMUs for ankle movements, a functional movement protocol was generated. Along with the reflective markers, two wearable IMU sensors were attached to participants. Participants were asked to perform a running task. Initially, they were instructed to naturally walk to synchronize the position of the markers and sensors as the zero spots and then to try running. The peak point (maximum dorsiflexion (Max DF) to Max DF) of this movement was detected to synchronize the two systems. Participants performed the running task. The data recording protocol consisted of five trials of running (2.68 m/s). Prior to the test, all participants were given time for a 10 min warm up and familiarization session, and they were asked to have a rest of 2 min between each trial.

### 2.7. Data Processing and Statistical Analysis

The motion capture data were considered the gold standard reference for kinematic data for this study. Data from the IMUs and VICON were synchronized by matching them based on the positive peak of the measure by each system [17]. The marker trajectories were imported to Matlab, and joint angles were computed and filtered with Matlab. The five cycles from Vicon and IMUs were synchronized using the positive peak value in the sagittal and frontal planes. The raw data were filtered by a fourth-order Butterworth low-pass filter with a cut-off frequency of 6 Hz, following the recommendation of previous studies [18], to attenuate unwanted noise. Data analysis was performed in Matlab software for running for both sagittal and frontal planes of movement. All data were calculated as averages of all repetitions before being averaged across all participants. All statistical analyses were conducted using SPSS ver. 25.0 (IBM, Armonk, NY, USA). For the test–retest, the intraclass correlation coefficient (ICC) was calculated for each plane of the ankle joint during running for each of the two systems [19]. Pearson‘s coefficient correlation was performed to verify the relationship of the ankle angle between IMUs and VICON in the sagittal and frontal planes.

## 3. Results

### 3.1. Demographics and Description

Ten male participants (mean ± standard deviation age: 30.2 ± 5.3 years; height: 171 ± 15.3 cm; body mass: 73.6 ± 12.4 kg) were enrolled in the study. Confirmed consent forms were given from all the participants. A total of 50 trials (running task; five trials per subject) were conducted and analyzed.

### 3.2. Reliability (Test–Retest)

The test–retest reliability of the IMUs in measuring the sagittal and frontal planes with ICC, and its standard error of measurement (SEM), is described in Table 1. A high correlation with ICC (2, 1) values of 0.974 and 0.9 for the sagittal and frontal planes were observed, respectively.

### 3.3. Validity (Pearson’s Coefficient Correlation)

**Figure 3:**
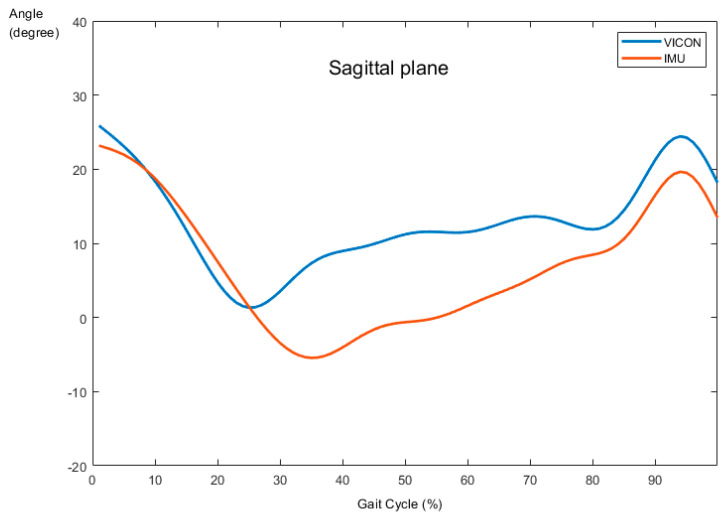
Comparison of ankle angle between VICON and IMUs in the sagittal plane.

**Figure 4:**
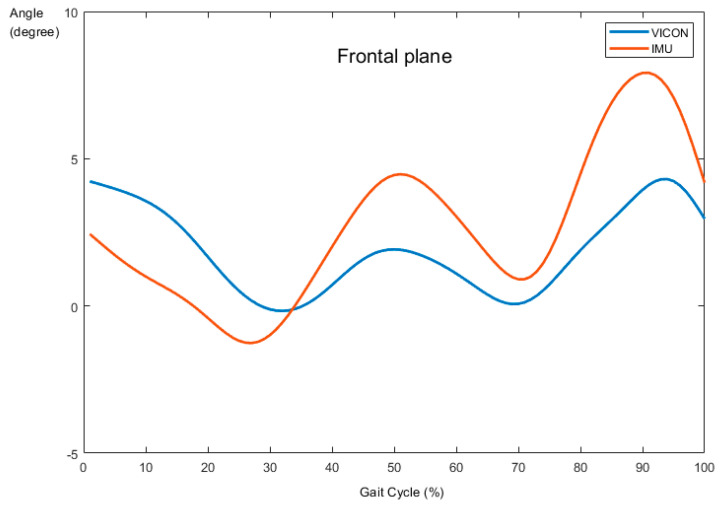
Comparison of ankle angle between VICON and IMUs in the frontal plane.

The validity test for ankle dorsiflexion/plantarflexion and eversion/inversion is shown in Table 2. Figure 3 and Figure 4 present the sagittal and frontal angles obtained from VICON and IMU systems during the running task, respectively. All planes showed high validity between the pattern of sagittal (*r* = 0.821, *p* < 0.001) and frontal (*r* = 0.835, *p* < 0.001) angles provided by the two systems.

## 4. Discussion

The primary aim of this study was to validate IMU measurement in the sagittal and frontal plane joint kinematics with the VICON system during running. The newly developed IMUs showed excellent reliability between the test and re-test measurements (ICC = X; 0.974, Y; 0.9). The ICC values for kinematic parameters were generally higher or equal to those in other studies, which only assessed the reliability during simple planar movements, such as the sagittal plane [12,15,20,21,22]. In addition, the validity described as a correlation of the joint angles measured by the two systems was significantly high in the sagittal plane (*r* = 0.821, *p* < 0.01) and frontal plane (*r* = 0.835, *p* < 0.01) during running.

Previous studies have reported a moderate to high validity of IMUs for measuring simple movement but showed low and varied to moderate values for complex movements, such as jumping and running, which may be because the complexity of the movements causes a problem for devices in transmitting and receiving data. Previous studies have suggested that utilizing Blooth, having the frequency-hopping spread spectrum function [23], and low speed transmitter of Wi-Fi [24] might cause technical problems which finally result in lower validity of IMUs in measuring the ankle joint kinematics during fast and complex movements. In this matter, we adopted a direct sequence spread spectrum with Wi-Fi and increased transmitting and receiving speed in our device. Although many efforts to improve the accuracy of data transmission functions of sensors have been made, the limitation of the place where it could be applied persists. However, with Raspberry Pi, the function of acquiring and saving a rapidly varying time-signal with high frequency [25,26], the system-on-chip, which enables the device to save data to its memory room, made it possible to be used in various outdoor activities.

In terms of the validity related to the specific movement—running, in this study—we chose the conventional gait model (PiG) to investigate the relationship between the newly developed device and VICON. One possible limitation of the proposed model is the different location of marker placement on a calculated joint angle, which was used to define the internal and external rotation of the tibia against the line of the ankle joint center, which could cause appreciable errors in ankle joint kinematics, especially the frontal and transverse planes [27]. In this regard, we devised a similar environment as a marker-based system to reduce errors between devices. The sensors were positioned perfectly parallel to each other, as well as the ground, by using a mini-inclinometer for accurate calculations. The results showed that IMUs seemed to be a suitable alternative to motion capture systems in both dorsiflexion/plantarflexion and eversion/inversion movements at the ankle joint during the running task [X: 0.821, Y: 0.835].

High accuracy for assessing the ankle joint movements in the sagittal and frontal planes was a different result from other previous studies [12,22]. According to the previous studies, the poor correlation between VICON and IMUs in measuring inversion and eversion was higher than dorsiflexion and plantarflexion due to its smaller range of motion. Specifically, they reported that if the complexity of movement increases, validity would decrease. In addition, they used only simple planar movement protocols, such as isolated flexion-extension, which may limit the generalizability of their conclusions. Our results extend these previous findings by considering a more challenging task: running.

As complex movements occurred at more than a single plane and with irregular movement velocities affecting system performance [28,29], an accurate method for proper calibration (proper alignment of the IMU axes with the anatomical segment axes) is considered as an essential factor contributing to reliability due to different calibration protocols may potentially result in substantially different consequences [30]. IMUs have been suggested as an alternative tool to the 3D motion capture system, because it provides real-time data in functional tasks within the same error range compared to classical measurement devices. Providing convenience to clinicians in kinematic measurements, it may be useful in clinical settings. Pathological patients such as cerebral palsy, Parkinson’s disease, and stroke have been shown to have ankle joint problems during gait, meaning that the analysis of ankle kinematics may be important in prescriptions. Our study showed the high validity of IMUs in measuring the ankle joint (against to VICON), showing the tendency of changes in the degree of the ankle. Therefore, our newly developed device may be useful for clinicians to detect the dysfunctions of their patients.

We acknowledge that our study has two limitations. First, as our sensors could not be rigidly fixed to the shank, a difference in the ankle joint degree between IMU and Vicon occurred due to the fluctuation in sensor motion. Second, although the possibility of application to the measurement in clinical settings has been suggested, we conducted the validation study with healthy individuals.

The development of device will enable us to provide valid data to assess the range of motion and joint orientation, and therefore, rehabilitation research and healthcare services will benefit from IMUs. Although more time and technical resources may be required from users to assess the patients until the system becomes more user-friendly, it will offer convenience with higher accuracy of kinematic measurements in clinical settings. We conducted our validation study with healthy subjects to reduce the error of validity; yet, IMUs need to ultimately benefit pathological populations and clinicians by guiding the clinical decision-making [31]. Therefore, in a future study, special considerations will be needed in pathological populations, as most calibration procedures require specific posture or movement [32].

## 5. Conclusions

We developed a system to measure the ankle joint angle using IMU sensors that are concurrent, convenient, inexpensive (approximately USD 300), light, and portable. Furthermore, it has a function of communicating with a computer via Bluetooth, and the computer is able to immediately calculate the data with Python. In order to validate the device, we compared the ankle X and Y angles data obtained from the IMUs with those acquired from the VICON system. The result of the comparison indicates that the IMUs and motion capture systems deviated the level precision to those well below normal measurements performed in a clinical setting. In the future, we will extend this approach to the pathological populations and, thus, apply it to the IMU-based training that provides multiple body joint angle kinematics in real time.

## Figures and Tables

**Table 1:** Intraclass correlation coefficient and SEM of VICON and IMUs for each plane.

Static Measurement	Sagittal Plane (ICC)	Frontal Plane (ICC)	SEM
VICON	0.978	0.969	0.39
IMUs	0.974	0.9	4.89

ICC: intraclass correlation coefficient; SEM: standard error of measurement.

**Table 2:** Pearson’s coefficient correlation of sagittal and frontal planes (IMU-based system).

Measurement	Sagittal Plane	Frontal Plane
VICON vs. IMUs	0.821 **	0.835 **

** *p* < 0.001.

## Data Availability

Not applicable.
